# Exploring first‐time mothers' experiences and knowledge about behavioural risk factors for stillbirth

**DOI:** 10.1111/hex.13662

**Published:** 2022-11-23

**Authors:** Tamara Escañuela Sánchez, Karen Matvienko‐Sikar, Sarah Meaney, Keelin O'Donoghue

**Affiliations:** ^1^ Pregnancy Loss Research Group, Department of Obstetrics and Gynaecology, Cork University Maternity Hospital University College Cork Cork Ireland; ^2^ Infant Centre University College Cork Cork Ireland; ^3^ School of Public Health University College Cork Cork Ireland; ^4^ National Perinatal Epidemiology Centre (NPEC) University College Cork Cork Ireland

**Keywords:** antenatal education, risk factors, stillbirth awareness, stillbirth prevention

## Abstract

**Background:**

Modifiable factors such as substance use, lack of attendance at antenatal care, overweight or obesity and sleeping position are associated with a higher risk of stillbirth. This qualitative study aimed to explore women's experiences of modifiable factors during pregnancy and their awareness of stillbirth.

**Methods:**

Purposive sampling was implemented by hospital staff in a large tertiary maternity hospital in Ireland between November 2020 and March 2021. Women were approached during their stay in the hospital and were invited to participate in a semistructured interview 3–5 months later. Eligible women were primiparous, >18 years of age and had an uncomplicated pregnancy and delivery. Eighteen women who consented to be followed up were interviewed at 3–5 months postpartum. Thematic analysis was used to analyse the data.

**Results:**

Four themes were identified: attitudes towards behaviour change, awareness regarding stillbirth and risk factors, the silence around stillbirth and risks, and attitudes towards receiving information about stillbirth. Women spoke about behaviour change in terms of outcomes, and most changes (e.g., ceasing alcohol consumption) were perceived as easy to manage. Awareness of stillbirth was limited among the women interviewed, and the association between risk behaviours and stillbirth was not known by any woman. Results suggest that there is a silence around stillbirth, including in antenatal care, which hinders information provision. However, most women highlighted the value of receiving information and extra education about modifiable risk factors and stillbirth.

**Conclusion:**

There is a general lack of understanding of the link between behavioural risk factors and potential pregnancy outcomes such as stillbirth. Providing further information to women about stillbirth and providing additional support with behaviour change might contribute to enhancing preventive efforts.

**Patient or Public Contribution:**

Patients were involved in this study by providing their experiences of antenatal care which were used as primary data.

## 1 INTRODUCTION

Stillbirth is one of the worst outcomes pregnant women and their families can experience.^1^, ^2^ Worldwide, the estimated average rate of stillbirth (defined as a baby being born with no signs of life at 28 weeks gestation or more) in 49 high‐income countries is 3.5 per 1000 total births, with rates varying from 1.3 (Iceland) to 8.8 (Ukraine) depending on the country.^3^ In Ireland, according to the latest report published by the National Perinatal Epidemiology Centre, the stillbirth rate was 4.06 per 1000 births in 2019, which reflected a slight increase compared with 2018 data.^4^ The overall perinatal mortality rate has remained flat in Ireland for several years, as opposed to the decrease observed in the decade before 2012.^4^ Although the rates of stillbirth in high‐income countries have reduced in the last 20 years, the variation between rates in high‐income countries shows that a further reduction of stillbirth incidence is possible and needed.^5^

Previous research has associated different types of risk factors with an increased risk of stillbirth, including medical,^6^, ^7^, ^8^ behavioural factors^9^, ^10^, ^11^ and sociodemographic factors.^12^, ^13^, ^14^, ^15^ Some of these risk factors are potentially modifiable and addressing them could contribute to reducing the rates of stillbirth.^16^, ^17^ Previous research has associated substance use including smoking, alcohol use and illicit drug use with an increased risk of stillbirth. Flenady et al.^18^ and Marufu et al.^19^ conducted two separate meta‐analyses examining the influence of smoking on the risk of stillbirth, and both concluded that smoking was associated with a 36% and 47% increase, respectively, in the risk of stillbirth.^18^, ^19^ Regarding alcohol consumption, Aliyu et al.^10^ concluded that mothers who consumed alcohol while pregnant were 40% more likely to experience stillbirth as compared with nondrinking mothers. Additionally, a recently published study exploring the risk of stillbirth in women who consumed alcohol and smoked in the antenatal period concluded that the adjusted relative risk for all stillbirths was 1.75 (98.3% confidence interval [CI], 0.96–3.18) for dual exposure, 1.26 (98.3% CI, 0.58–2.74) for drinking only and 1.27 (98.3% CI, 0.69–2.35) for smoking only compared with the reference group.^20^ Being overweight and obese has also been associated with an increased risk of stillbirth. A recent study exploring prognostic variables for stillbirth found that the most frequently reported maternal characteristic associated with stillbirth was high BMI and other measures of obesity, and these findings were supported by highly convincing evidence in most of the studies included in their review.^21^ Engagement with and attendance at antenatal care has also been linked to the risk of stillbirth. Stacey et al.^22^ concluded in their study that attending less than 50% of the recommended visits was associated with an almost three times increased risk of stillbirth, and this risk increased as the number of visits attended decreased. Last, women's sleeping habits have also been studied in association with stillbirth risk and research has specifically focussed on the maternal sleep position. Cronin et al.^23^ conducted a meta‐analysis which concluded that the supine going‐to‐sleep position is independently associated with late stillbirth.

Even though there is evidence in the literature associating these modifiable risk factors with stillbirth,^16^ information about the role of these behaviours in stillbirth is not widely available amongst the public in Ireland, as demonstrated by Nuzum et al.^24^ in their survey study. These authors, after surveying 999 members of the Irish population, reported that there is a lack of public awareness of the incidence, causes and risk factors for stillbirth, although over 50% of respondents personally knew someone who had experienced a stillbirth.^24^ These findings are similar to those obtained in a survey study conducted among members of the American College of Obstetrics and Gynaecology.^25^ The authors concluded that knowledge regarding epidemiology, risk factors and effective interventions to reduce stillbirth amongst participants was only fair. In the same study, only 30% of respondents were aware that preeclampsia, advanced maternal age, elevated α‐fetoprotein, multiple gestations, cigarette smoking, illicit drug use and being postterm increased risk.^25^ Regarding knowledge of behavioural risk factors, previous studies have also demonstrated that some women held serious misconceptions about the risks of substance use or engaging in weight management behaviours, as well as having limited reproductive knowledge, which interferes with their care‐seeking behaviours and increases their risk of stillbirth.^26^, ^27^, ^28^

Previous research has also shown that stillbirth is still a taboo subject in society.^29^ Hence, further efforts to increase women's awareness about stillbirth and its risk factors through routine antenatal care and public health campaigns are necessary to support stillbirth prevention efforts.

Involving patients' experiences in designing and developing public health campaigns and behaviour change interventions is vital to ensure that they are tailored to users' needs. Previous research has established that women engage in behaviour change during pregnancy due to different motivations, but the volume of expectations placed on them and the complexities of those changes are rarely acknowledged.^30^ Understanding women's experiences of behaviour change and information provision during antenatal care through their perspective is essential to inform the development of such interventions and public health campaigns. The main objective of this study was to explore women's experiences of modifiable factors during pregnancy and knowledge and beliefs regarding behavioural risk factors related to stillbirth. Additionally, we aimed to examine women's experiences, if any, of being informed about such risk factors during their antenatal care.

## 2 METHODS

To enhance the reporting of this study, the Standards for Reporting Qualitative Research (SRQR) checklist has been used^31^ (see Supporting Information: File 1).

### 2.1 Design

A qualitative semistructured interview study was conducted using a Reflexive Thematic Analysis approach (see Supporting Information: File 2). Qualitative research allows the researcher to explore and understand social phenomena and psychological concepts such as experiences, beliefs, motivations and attitudes.^32^ For this study, a constructivist paradigm was utilized.

Constructivism is based on the idea that knowledge is built from an interaction between the subject and reality, and hence, the same situation experienced by different people might result in different interpretations, depending on each individual's perception of such a situation.^32^ This epistemological approach refuses the idea that there is only one truth but instead supports the importance of taking into consideration the subjective meaning given to a phenomenon and the understanding of the context of the same.^32^ In constructivism, the researcher and the study participants are equally involved in the process to generate knowledge, hence, the researcher has an active role in the research processes by creating—rather than revealing—something which has to make sense within existing frameworks of meaning.^32^

This paradigm allowed us to understand the complexity of our participants' lived experiences from their point of view.

### 2.2 Recruitment

Women were recruited using a purposive sampling approach implemented by hospital staff in Cork University Maternity Hospital (CUMH), Ireland. Inclusion criteria included primiparous women, with a low‐risk pregnancy and uncomplicated delivery, who had a healthy baby and were 18 years old or older and consented to participate in a one‐to‐one interview 3–5 months postpartum. The follow‐up timeframe of 3–5 months postpartum for interviews was chosen to facilitate women adapting to their new routine with their babies before taking up their time to participate in the interviews.

Exclusion criteria included multiparous women, women currently pregnant, women with a history of pregnancy loss, women attending high‐risk antenatal clinics, congenital anomalies, cases of maternal morbidity and cases of major complications during pregnancy or birth for both woman and baby. Given the aims of our study, it was decided that obtaining the experiences of primiparous women with uncomplicated pregnancies and deliveries would reflect the common practices regarding information provision and knowledge acquisition during antenatal care. It was assumed that including women with additional care needs during their pregnancy or delivery would have resulted in a biased picture of the normal day‐to‐day practices of the antenatal care services, as these women require additional education and are exposed to different types of healthcare professionals and levels of care.

Women were invited to participate in the study during their stay in the maternity hospital after delivering their babies from November 2020 to March 2021. If eligible, an obstetrician affiliated with our research group explained the study to the woman and provided them with the patient information leaflet and consent form. After providing women time to read the information and ask questions, their written consent was obtained by the obstetrician. The woman's contact details were obtained at this first encounter, and women were advised that a researcher would contact them 3–5 months later to participate in a one‐to‐one interview.

### 2.3 Setting and sample

CUMH is 1 of 19 maternity units in the Republic of Ireland and is situated within the south/southwest hospital group. In 2020, 7040 babies were delivered in the hospital of which 42.2% were born to first‐time mothers.^33^ Forty‐four women consented to be followed up 3–5 months postpartum in the current study. One researcher (T. E. S.) contacted all women who agreed to participate in the study between 3 and 5 months postpartum by phone or e‐mail. Of the 44 women, 4 subsequently declined to participate and 22 were unreachable or did not attend the scheduled online interviews. Eighteen women subsequently participated in the individual semistructured online interviews between February and July 2021.

In Ireland, even though access to maternity services is free, the option of choosing private or semiprivate care also exists. All women participating in this study gave birth in CUMH. Some participants used the service as public patients, meaning all their antenatal care was provided by CUMH staff; other women used the service as private patients, meaning that their antenatal care was mostly provided by a specific clinician's private practice staff.

### 2.4 Data collection

Women were interviewed about their experiences of behaviour change on modifiable factors during pregnancy and information provision regarding stillbirth and modifiable risk factors for stillbirth during their antenatal care between February and July 2021. Online one‐to‐one interviews using Google Meet were used as the data collection method due to the restrictions imposed on in‐person data collection during the COVID‐19 pandemic. The women participating in the study were sent a link to a scheduled online Google Meet at the date and time of their choice, with instructions on how to access the online meeting. The researcher's contact details were also provided to be used in case of technical difficulties.

All online one‐to‐one interviews were conducted by T. E. S. The semistructured interviews were guided by a pre‐agreed topic guide developed by the multidisciplinary team and informed by previous work of the research group.^26^, ^27^, ^28^ The topic guide included the following areas: history and health habits, awareness of risk factors for stillbirth, feelings and opinions about receiving education on stillbirth, information sources and interventions (see Supporting Information: File 2). Some of the questions in the topic guide were designed to elicit information about specific topics such as knowledge about stillbirth and risk factors associated with stillbirth. The use of semistructured interviews allowed the women to introduce or discuss topics that were not strictly predefined in the interview topic guide. As the interviewing process progressed reaching the 14th and 15th interviews, it was clear that the depth and quality of the interviews indicated that it was not necessary to continue actively pursuing the nonresponders to the invitation following their initial consent. It was then considered that enough information power was achieved. The concept of information power is proposed by Braun and Clarke as an alternative to the concept of data saturation which they deem incompatible with Reflexive Thematic Analysis. The concept of information power relies on the fact that rich, relevant data requires fewer participants,^34^, ^35^ hence the more relevant information a sample holds, the fewer participants are needed.^35^ However, all 18 women for whom an interview was scheduled were interviewed to respect the commitment that had been made with them.

Interviews lasted between 30 and 50 min, were recorded, transcribed verbatim and imported into NVIVO 12 for analysis.

### 2.5 Data analysis

The data analysis conducted in this study is based on the principles of Reflexive Thematic Analysis as described by Braun and Clarke.^32^, ^36^ Reflexive Thematic Analysis is a flexible method that is suited to experiential and critical framings of language, data and meaning, and it can be used in either a deductive or inductive way. Reflexive Thematic Analysis involves an interpretative reflexive process, coding does not follow a framework and themes are the outcome of the analytical process.^37^ We conceptualized reflexive thematic analysis for this paper within a constructivist approach (see Section 2.1).

The different phases of thematic analysis described by Braun and Clarke are as follows: (1) data familiarization and writing familiarization notes; (2) systematic data coding; (3) generating initial themes from coded and collated data; (4) developing and reviewing themes; (5) refining, defining and naming themes and (6) writing the report.^32^ The analytical process in the current study began by transcribing the audio of the recorded interviews (data familiarization), to facilitate this process, Tactiq was used during the interviews, which is an automatic transcription tool. One researcher (T. E. S) read and reread all of the interview transcripts. Inductive open coding was then initiated which facilitated the identification of units of meaning that related to the research aims. Subsequently, those codes were categorized and grouped into themes and relabelled where appropriate. A record of the evolvement of the themes and the category names was always kept. Further analysis allowed the researchers (T. E. S. and K. M.‐S.) to group the different categories into themes, by refining their meaning to portray the story the data tells. A second author (K. M.‐S.) reviewed and followed the coding process at all stages, and discussions were held as necessary.

### 2.6 Ethical considerations

The clinical team who obtained consent from women to participate in the study ensured that the sensitive nature of the interview topic was discussed to anticipate potential distressing factors for the women. Women were informed about their rights to withdraw from the study at any stage without any potential impact on their care or any other type of consequences. We considered the potential for women to experience distress while participating in the interviews and so planned accordingly to provide support to women who experienced distress.

This involved ensuring that the researcher would remain in the online session with the woman after the interview and until she was happy to end the session. The researcher would then make a follow‐up contact if deemed necessary or contact the appropriate required support (e.g., referral to specialist bereavement midwives or perinatal mental health services). However, none of the participating women reported distress or were observed to experience distress and so this support was not provided to any participants.

### 2.7 Reflexivity statement

The research team that conducted this study includes experts in health psychology, epidemiology, behavioural science, public health and obstetrics and maternal–fetal medicine. All interviews were conducted by T. E. S., who is a female PhD student with a background in health psychology. T. E. S. received training in qualitative methods and interview techniques before conducting this study.

All three remaining authors are highly experienced researchers who have been involved in multiple qualitative studies.

None of the authors had a previous relationship with any of the participants, and the participant only had contact with the researcher conducting the interviews (T. E. S.).

The participants were informed that the interviewer was a PhD student and the overall objectives of the PhD project were exposed to them before commencing the interviews.

All members of the research team interacted throughout all phases of this study, which enhanced the process by providing points of view from different disciplines.

## 3 RESULTS

### 3.1 Sample characteristics

The final sample of 18 women included 16 White Irish women, 1 White Eastern European woman and 1 White North American woman. Woman's ages ranged from 28 to 37 years old. All women were married or cohabiting with their partners (see Table 1).

**Table 1:** Women's characteristics

Women number	Age	Insurance status	Occupation	Relationship status
W1	31–35	Public	Management	Married
W2	25–30	Private	Healthcare	Married
W3	>35	Public	Education	Cohabiting
W4	>35	Private	Social Care	Married
W5	>35	Public	Retail	Married
W6	31–35	Private	Healthcare	Married
W7	31–35	Public	Social Care	Cohabiting
W8	31–35	Public	Management	Married
W9	31–35	Public	Management	Married
W10	31–35	Private	Management	Married
W11	>35	Private	Education	Married
W12	>35	Public	Hospitality	Cohabiting
W13	25–30	Public	Education	Cohabiting
W14	25–30	Public	Healthcare	Cohabiting
W15	31–35	Public	Healthcare	Married
W16	31–35	Public	Healthcare	Married
W17	25–30	Private	Engineering	Married
W18	31–35	Public	Management	Married

### 3.2 Findings

Several themes and subthemes were identified through the analytic process (see Table 2).

**Table 2:** Themes overview

Themes	Subthemes
Attitudes towards behaviour change	
Awareness regarding stillbirth and risk factors	Awareness regarding health advice
	Limited awareness about stillbirth
Silence around stillbirth and risks	Lack of discussion regarding stillbirth and risk factors
	Reliance on own information‐seeking behaviours
Attitudes towards receiving information about stillbirth	‘Knowledge is key’
	Stillbirth perceived as a difficult topic
	Importance of language and preference for information provision

#### 3.2.1 Theme 1: Attitudes towards behaviour change

All women expressed having made changes in their behaviours because of their pregnancies. In some instances, these changes started preconceptually in preparation for pregnancy and were maintained throughout the pregnancy. The preconception preparatory behaviours adopted by women involved attending their general practitioner (GP) for advice, adopting healthier eating behaviours and having a more active life, monitoring menstrual cycles, preconceptual alcohol abstinence, preconceptual intake of folic acid and antenatal vitamins, as well as prepregnancy weight loss. Women spoke about the changes they made as soon as they learnt they were pregnant, the most common behaviours being ceasing alcohol consumption, avoiding foods not recommended during pregnancy, taking vitamins and folic acid, staying active, moderating the intensity of physical activity, having a nutritious diet and/or increasing rest.

Women discussed newly adopting some of these behaviours to achieve their best health status (e.g., being as fit and eating as healthily as possible, taking folic acid), and also abandoning some previous behaviours to prevent illness and adverse outcomes (e.g., avoiding consumption of alcohol, avoid stress, quitting smoking, etc.). These results show that women perceive behaviour change during pregnancy to have a dual nature in that it can help to improve positive outcomes and reduce the likelihood of adverse outcomes.

I just took vitamins and I did go to the doctor, alright? For advice, because I was on the pill for so long and I was a bit worried (W11)

I used to drink, alcohol, at weekends, but I stopped. And I obviously stayed off as well while I was pregnant. (W6)

I was exercising intermittently and I was kind of prioritizing work and I just said ‘no I'm going to prioritize myself a little bit more’ so I made it my purpose to just do swims and run more often, that was it. (W15)

Women discussed their different experiences when engaging in behaviour change during pregnancy. All women in this study expressed a strong and clear opinion about the need to stop consuming alcohol during pregnancy. However, some women decided to stop consuming alcohol as soon as they started planning the pregnancy, while others waited until they had confirmation of pregnancy. Regarding smoking and illicit substance use, women also expressed negative attitudes. Only one of the women was a smoker before her pregnancy, and she quit as soon as she learnt about her pregnancy. Physical activity was perceived as beneficial to achieve an appropriate fitness level and general health status, rather than a tool to manage weight gain. Some women expressed difficulties keeping up with their levels of exercise through their pregnancy, which led them to slowly disengage from these behaviours in response to perceived challenges associated with pregnancy (e.g., lack of energy, nausea). On the other hand, the adopted changes in diet were mostly focused on avoiding foodborne diseases, with some women starting to relax their attitude towards their food consumption as the pregnancy progressed.

I think it was less about weight management and more about just fitness, or like wellbeing or health or whatever, more cardio and all that more so than the weight management thing. (W17)

Women had the perception that they had been able to continue with their normal life during their pregnancy with little interference from their pregnancies. Most of the women perceived their behaviour changes (e.g., stopping alcohol consumption, avoiding dangerous foods, engaging in low‐demand physical activity) as easy to manage and natural because they were doing these changes for their babies. However, one woman expressed after the interview that actively thinking about all of the changes made during her pregnancy helped her realize that she had made more changes than she had previously perceived. Hence, it seems that the fact that women perceived all their behaviour changes as natural might have contributed to the fact that they were less consciously aware of the range of different changes they did engage in. Women also discussed behaviour change during pregnancy in terms of consequences for the health of their babies or their own health which acted as their drive to change their behaviour. As an example, several women spoke about exercise as useful to facilitate labour in general.

I thought that the having no drinks would be harder than it was because I'd never been one to be able to go months and months without drinking. But it was actually easier than I thought because I was doing it for my baby. (W18)

Yes, I had the day sometimes that I really wanted to smoke but, you know, because I was waiting so long for that baby. I was like, ‘no, I'm not gonna put her in any risk no matter what’. (W12)

it's all actually just coming back now talking. I'm like, ‘no, I didn't really change much’. Yes, I actually did. (W5)

#### 3.2.2 Theme 2: Awareness regarding stillbirth and risk factors

##### Awareness regarding health advice

Most women in this study were aware of the importance of maintaining a healthy diet and adequate levels of physical activity during their pregnancies, with little concern about the safety of the physical activity. Women were also aware of the relevance of antenatal vitamins and prepregnancy supplements, especially folic acid.

I just always knew that if I did want to fall pregnant that I should be on folic acid. I really started ensuring that I took it for the six months beforehand, but I've kind of really been taking it for years sometimes. (W10)

Risks associated with substance use were also discussed by many of the women. For instance, women were aware of the recommendations regarding alcohol consumption during pregnancy, and all women in this study decided to abstain from alcohol. All of the women also discussed smoking as a behaviour with risks for the baby. Although alcohol consumption and smoking were issues mentioned by almost every woman, some women also commented on the risks associated with illicit drug use.

I was at a party and I was said, like, ‘you know, one drink is okay for the baby’ but I was reluctant. (W15)

Um, obviously smoking is a big no‐no or any drugs of any description. (W9)

Some women were also aware of the risks associated with sleep position and were aware of the importance of monitoring their baby's movements, which for one woman was a source of distress.

##### Limited awareness about stillbirth

Women were explicitly asked about their knowledge of stillbirth and the risk factors associated with stillbirth. Our findings suggest limited awareness about stillbirth among the women included in this study. Some women discussed an understanding that stillbirth is a pregnancy loss that can occur later during pregnancy. Further, most women openly expressed that their knowledge about stillbirth and related risk factors was very limited. When explicitly asked, none of the women reported that information about stillbirth was received from a healthcare professional during their antenatal care.

No, I think I have a very limited view of it. You know that baby is born and unfortunately, baby is not born alive but I wouldn't say that I know the reasons why are … you know, obviously it was something that crossed my mind […] but no, I can't say that I know a whole deal about it. (W3)

I suppose what I know about stillbirth is when a baby is born and they're dead. I know that there are stillbirths were you can go through full pregnancy and you can give birth and you don't know that your baby's not going to breathe when they come out, and that's just so sad. Then I do know that there's others where a baby may … their hearts might stop or they might stop breathing during pregnancy and you know that and then you have to give birth to the baby, you know? And they're … that's kind of as much as I know really. (W10)

Additionally, some women confused the term stillbirth with other adverse outcomes given their lack of knowledge of the definitions of pregnancy/infant loss. Women were not aware of the differences between the concepts of stillbirth and miscarriage regarding the gestational cut‐off point that differentiates them, and some women conceptualized stillbirth as an event that can only occur during labour. Further, two women confused the term stillbirth with Sudden Infant Death Syndrome.

I'm not sure, can it happen at any stage during pregnancy? can the term stillbirth and miscarriage, you know, can they be used in the same? (W8)

I thought it was kind of random…, that it was most risky before kind of 20 weeks was the highest probability [of experiencing stillbirth] or whatever. Um, that's all I really know. (W17)

I don't know a huge amount about it, but it's if a baby unexplainably passes away and usually, when sleeping before they're six months and the child can seem to be perfectly healthy and there doesn't seem to be an explanation for it. (W4)

Most of the women in this study expressed that they were ‘guessing’ or ‘supposing’ when asked what their knowledge was about stillbirth and risk factors. Some women stated their knowledge and awareness were drawn from other people's experiences of pregnancy loss in their social circles (e.g., friends of friends, distant relatives and neighbours).

The behaviours that women thought were most likely related to stillbirth were substance use, sleeping on their back or doing exercise lying down on their back, or ‘knocks on the stomach’. Women also spoke about and identified behaviours such as substance use or consumption of certain foods associated with other adverse pregnancy outcomes (e.g., long‐term developmental delay, physical disabilities or extreme prematurity); however, the link between such risk behaviours and stillbirth was not present in the majority of women's discourses. These other potential adverse outcomes were perceived as very relevant, concerning and more present in women's minds during pregnancy than the possibility of stillbirth.

I think most women are more concerned with developmental issues with like the drinking and smoking and things like that. They are more thinking. ‘okay, when my baby comes out is it going to have learning issues?’ No one actually thinks about stillbirth. (W18)

I didn't actually worry about stillbirth, I worried about early miscarriage, I worried about falling like that kind of stillbirth, maybe an early delivery and extreme prematurity and neonatal units. (W6)

#### 3.2.3 Theme 3: Silence around stillbirth and risks

##### Lack of discussion regarding stillbirth and risk factors

Many women felt that they had not received information about stillbirth, health habits or risk factors for stillbirth during their antenatal care. When asked, none of the women could remember a moment during their interactions with healthcare professionals (e.g., midwives, consultants) in which they had received information about stillbirths.

It is kind of common knowledge, you know, ‘don't drink. Don't…’ you know, like a lot of it, I think people kind of know already. (W13)

Regarding specific health habits, such as physical activity, women perceived that healthcare professionals adopted a very conservative approach towards offering advice, and the general message given to women was to ‘not overdo it’. Regarding weight gain, women also expressed a lack of advice in their interactions with healthcare professionals, and most women reported only being weighed at the time of antenatal booking.

No, no, not really [got any advice regarding physical activity]. Of course, that's if you did some like, very hard Exercise, you have to stop a bit so they warned me about it. (W12)

On the other hand, some women reported receiving some information regarding health habits and risk factors (e.g., keeping physically active, avoiding substance use, sleeping on the left side or monitoring the baby's movements), even though this information was never associated with the risk of stillbirth. In these cases, the information would normally be provided by the women's GP, the hospital midwives or the midwife at their consultant's private clinic.

They were both lovely [Consultant and midwife] and so Midwife would have done a lot of the antenatal education when I went in to see her before I went to see Doctor. (W2)

That's something I came across in my public health nurse, she said everything. And she did say to me, ‘sorry, if I'm being kind of condescending or telling you things, you know, but I need to tell you in case you don't know’. (W17)

The information that women discussed being provided by healthcare professionals regarding risk factors and health habits was mostly focused on sleep position, monitoring fetal movements and preventing food‐borne diseases. However, even though some women were provided with this information, in most cases women reported that they were not informed about the reasons to adopt these behaviours. Most women received written information during their care, and even though they valued it and used it to prompt discussions with their providers, they did not feel it could replace a conversation with a healthcare professional.

It was just a brief conversation that was had in the earlier stages [about left‐side sleeping]. And I don't even know why. (W15)

I was advised that if I felt any reduced fetal movement […] I presumed that was down to … chances of stillbirth or being at risk of something happened in the baby. This was all after 24 weeks and I know that stillbirth is after 24 weeks. So, I presume, I just put that down to being that without ever mentioning this is a risk of stillbirth. Nobody ever mentioned those words to me in that time. (W15)

According to women's accounts, it seemed that the limited information provided by their healthcare providers might relate to the fact that the participant women were healthy and were not experiencing complicated pregnancies.

I don't feel like they felt the need to have that conversation with me because I wasn't part of the criteria for it perhaps. (W15)

I suppose the fact that I was quite young, the fact that I was low risk and I had no other health concerns … It wasn't the thing that they really discussed with me. [.] So we were happy out and I didn't have any signs or symptoms to suggest that there might be something wrong. (W2)

##### Reliance on own information‐seeking behaviours

Most of the women who participated in our study reported feeling able and confident when trying to find information about their pregnancies using information sources of their choice (e.g., websites and books). Our findings show that most of the awareness that women showed about stillbirth, risk factors and health habits during pregnancy was a consequence of the women's autonomous information‐seeking behaviours. Information‐seeking behaviours sometimes translated into decision‐making modulating women's behaviour. However, in other instances, finding conflicting information (e.g., in online forums) acted as a source of concern.

I was happy with that because I knew myself that I got my information from a variety of sources […] I was kind of happy with my own research. (W16)

I was actually looking and I found like different information because there are some pages were saying that you should sleep on the left side, some people on the right side, so I was wondering, should I sleep on the left side or the right side? (W12)

Proactive information‐seeking behaviour led some women to feel that they already had enough information through their research, and they did not require additional professional advice. However, some women acknowledged that only relying on the information provided by their healthcare professionals would have made them feel uninformed.

Like it was fine, I'm well able to kind of Google things and research myself. (W9)

I don't think so [that information provision would have been enough without her own research]. Because I have a friend who refused to do any research. She was just totally caught off guard. With lots of different things that happened, yeah, I don't think there was enough information there but I mean maybe they would have given me more information if they got the impression that I didn't know, you know. (W16)

Women also spoke about some of the characteristics of the sources of information that they used during their pregnancies. Women expressed a strong reliance on official sources, like websites or hospital‐provided books, and had a critical attitude towards certain sources of information, especially those found online.

I did take on board what was in the HSE websites […] I did find the HSE website quite trustful as well. (W2)

Most of the information I just got from the HSE book, I found the HSE book actually to be excellent. That was probably the best, along with the nurses and, and the consultants, that was one of the best sources of information. I just kind of stuck to that because I it's accurate what was in it. (W4)

Some of the sources of information named by the women were: hospital‐provided books, commercial mobile applications, websites, family and friends, conversations with healthcare professionals, social media, online antenatal classes, formal education, peers and podcasts. Most women engaged with commercial mobile phone applications (apps) to obtain information, and expressed that one of the features that they appreciated the most about apps in particular was the weekly updates and notifications; these features provided women with timely relevant information for their stage in pregnancy.

[weekly updates] it helped me along the journey. Kind of nearly makes it easier, you're better with something that you can see, now you know you're getting bigger, but like you can kind of just see the journey and it helps you along, but it was just really simple. (W10)

#### 3.2.4 Theme 4: Attitudes towards receiving information about stillbirth

##### ‘Knowledge is key’

Eight women in our study had a positive and open disposition towards receiving information about risk factors and stillbirth during their antenatal care from their healthcare professionals. For these women, information was perceived as a tool that might have an influence on preventive efforts, and also facilitate the grieving process of parents who experience stillbirth.

I think it is good to know about stillbirth because I think it's a very Irish thing maybe, that we don't talk about things that we don't want to talk about. It should be spoken about. I think we should be told about it. We're told about everything else. Then, you know, if there's risk factors for stillbirth and it's also risk factor, we should be made wherever of it. A lot of people don't talk about it. There's a stigma around it, I understand why. It's, you know, it's sad, it's heart‐breaking. (W10)

I think to have the knowledge would be way more beneficial than negative. Even if I did go away with a little bit of concern about it, I think that's very natural because you fear for your baby and you want to do everything that you can. But I think overall it would have been extremely positive. (W15)

It's better to be prepared basically for the unexpected. (W5)

Some comments made by the women indicate that women's concerns regarding pregnancy loss are focused on the first trimester and that the probability of loss is almost nil afterwards. This is a popular misconception that might have been supported by the women's family or social context.

I suppose with stillbirth it's the same kind of thing, preparing women that this is not a ‘You've now reached 12 weeks it's not a 100% guarantee’ and I suppose if you have no awareness of it and it happens to you would be so shocking. (W18)

Some women felt that receiving information about stillbirth and its modifiable risk factors would also work to facilitate women's decision‐making, by providing them with enough information to facilitate making informed judgements about their risks.

I think information is power. Just to give them as much information as possible and then they eventually choose what they want to do with it. But at least they have the information then. (W9)

##### Stillbirth perceived as a difficult topic

Most of the women participating in this study engaged in a process of balancing the pros and cons of receiving this information, which was evident in their discourses. Some of the women in the study expressed that they would have found stillbirth a difficult topic to discuss, especially during pregnancy. However, some women with this opinion also recognized that stillbirth was a latent concern of theirs during their pregnancies.

It was something I didn't want to talk about and I wouldn't have brought up myself, but if they had approached me with this, I would have been absolutely fine to talk about it. (W3)

When you're pregnant, it's something you tend to not want to overly research just because […] you're hypersensitive at that time also. (W8)

Nine of the women expressed that discussing the topic of stillbirth might have the potential to increase their levels of anxiety during pregnancy. The word stillbirth was perceived as a societal taboo which, for some women, acted as a barrier to both information seeking and information provision. However, all of these women felt that the balance between being empowered with the information, despite having certain negative feelings, was still positive. Women rationalized their decision by considering the information as a positive resource, but they highlighted the importance of tailoring the information based on each woman.

I think it would have been counterproductive, but you know … saying that I'm a person who likes to know all the facts. So, you know, I would like to think that if I did ask that they would have been very forthcoming and very honest with the information that it wouldn't be a case of ‘oh you don't need to worry about that’. (W8)

I think knowledge is key so I would have absorbed the information and taken on board for sure. However, being and discovering that I'm a bit of a worrier, there's no doubt it would have played in my mind. (W15)

I think it would be beneficial, but I'm not sure everyone would want to hear it. Like if you're quite emotional and it is an emotional time, but I do think it is good to kind of maybe touch on it and maybe not put too much emphasis on it. (W9)

One woman had a negative attitude towards receiving information about stillbirth and risk factors, for this woman, the benefits of obtaining the information did not justify the potential harm.

If someone just says ‘don't drink alcohol’ fine. You don't do it. If someone says ‘don't drink alcohol because you might have a miscarriage’ and then you're worrying about it … Does worrying increase the risk? I don't know. No, it's probably better that it's not pointed out maybe, for fear that you do worry more about it and you worry unnecessarily. (W17)

##### Importance of language and preferences for information provision

Some of the women that participated in our study insisted on the importance of the language used when providing information about stillbirth and risk factors. Women felt that the best way to provide education about stillbirth and risk factors was to do so in a very sensitive way and avoid the use of ‘blaming’ vocabulary when talking about the behaviours that might increase the risk of stillbirth.

So while it might invoke some degree of upset. I think if it's done in a sensitive way and in a kind of a positive slant of, you know, ‘we know there are some things that we can do to reduce this risk’, that I would think that most women would be receptive to that. (W6)

Maybe do it without using the word stillbirth: ‘avoid these behaviours during pregnancy’ or something. Yeah. (W13)

Maybe it's just the people don't like to say the words, you know? (W15)

Regarding women's preferences for information provision, women highlighted the importance of having reliable sources, and providing specific advice or information, with the option for more information on demand. Women also expressed a preference for group educational sessions on behavioural risk factors for stillbirth and best health habits for pregnancy. According to these women's opinions, receiving information about stillbirth and risk factors for stillbirth on a one‐to‐one basis would have made them feel as if there was something wrong with their pregnancy, whereas receiving the information in a group format would be considered as ‘just another topic to discuss’.

I think maybe groups, because I think if it's one on one, the person might feel it's being directed at them. Whereas if it's in a group, it's just, you're informing them. (W5)

When it comes to the best timing to provide women with information about stillbirth and risk factors, women felt that this information should be provided early in pregnancy. These women were aware of the importance of not engaging in risky behaviours as soon as possible, and so they considered that receiving the information early would help prevent adverse outcomes in pregnancy. Some women expressed that they had a heightened cautious attitude at the beginning of their pregnancies and that this could be taken advantage of in terms of increasing awareness about risk behaviours. Additionally, some women expressed the potential for conversations about stillbirth between women and healthcare providers to become more difficult as the pregnancy progresses, when women's concerns start focusing on labour and birth.

There's a lot of information at the start, but it's really that's when you need to be doing it [changing behaviour], […] I think giving the information earlier is better. (W15)

I think earlier in the pregnancy so they have time to set up for a healthier pregnancy. (W14)

However, other women considered that the information about stillbirth and risk factors should be provided in the second trimester since the first trimester represents a vulnerable period, in which discussion of stillbirth would constitute additional stress and pressure for the woman. Whereas other women, especially those with a background in healthcare, highlighted the importance of the preconceptual period as the best time to provide education to women.

Maybe in between 12 week and the anomaly can. I think a lot of people are kind of stressed about getting to the 12 weeks anyway […] I think maybe we should leave people get to that stage rather than stressing them out even more. (W1)

At the beginning I think there's enough stress anyway … after the half, if you know that everything is fine, that kind of information won't actually scare you so much like at the beginning. (W12)

## 4 DISCUSSION

Our findings have shown that information provision and awareness about stillbirth and associated behavioural risk factors are mostly poor amongst most women with uncomplicated pregnancies and births participating in our study. Women showed good awareness regarding health recommendations and behaviours to avoid during pregnancy, however, regardless of the level of knowledge that women expressed about stillbirth or potential behaviours associated with an increased risk of stillbirth, it seemed that there was not a clear link establishing the association between risk behaviours and stillbirth in women's discourses. Further, the awareness that women expressed regarding risk factors and stillbirth was predominantly a result of their information‐seeking behaviour. Women expressed that information provision during their antenatal care regarding health behaviours was poor, and information regarding stillbirth was nonexistent. Women in this study showed varying attitudes regarding being provided with information about stillbirth and behavioural risk factors for stillbirth. Whereas some women perceived information as a tool to improve prevention efforts, other women considered that this information was not necessary in all cases and it would only increase anxiety levels for them.

Women's reasons for engaging in behaviour change were commonly associated with obtaining the best possible outcomes for their babies and themselves. This motivation to make healthy choices and a sense of responsibility driven by the desire to improve the baby's health and reduce risk has been described in the extant literature.^26^, ^27^, ^28^, ^38^ Further, women's perception of risk, which can only be accurate with appropriate communication and awareness, will also influence their decision‐making.^38^, ^39^ This highlights the importance of providing accurate information to women about stillbirth related to different behaviours during pregnancy. Providing women with this information might increase their level of motivation to tackle modifiable behaviours and reduce their risk of stillbirth.

Women in our sample were able and willing to conduct their research and find answers to their concerns, and one of the most mentioned information resources was the internet. However, we know from previous research that the information available online regarding stillbirth and behavioural risk factors for stillbirth in Ireland and the United Kingdom is scarce and difficult to access.^40^ Further, women in our study were a small number of predominantly highly educated White Irish women. Previous research has demonstrated that sociodemographic characteristics are associated with the choice of a particular source of information, and also with the number of sources used,^41^ with being female, educated and young the best predictors to engage in health information‐seeking behaviours.^41^ Therefore, it is possible that the positive attitude towards information seeking in our sample is not representative of the general population, and further research would be necessary to explore the needs of people from different sociodemographic backgrounds.

Previous research has demonstrated that the levels of knowledge and understanding that women have about advice received during pregnancy will have an impact on their psychological capabilities towards behaviour change.^28^ Women who are less aware, or who held misconceptions about the consequences of their behaviours will be less likely to engage in behaviour change.^28^, ^42^ The women in our sample had limited knowledge about stillbirth and its associated risk factors; however, when prompted to speak about the advice received during their antenatal care, monitoring of fetal movements and the importance of sleep position was often mentioned. These findings are very similar to those obtained by Stacey et al.^43^ in a recently published study, where they explored migrant women's awareness of health messages to reduce stillbirth risk. Our findings also highlight the importance of promoting health education during pregnancy from the healthcare professionals involved in women's care, as well as the value of generating additional written or online resources for women.

Additionally, our results show that women prefer antenatal groups to receive information regarding their health and their pregnancies, and most women found that antenatal classes represent appropriate spaces to share information about stillbirth and risk factors for stillbirth. This is positive considering that previous research has demonstrated that passive transfer of information is not sufficient to prepare women and their partners for birth and parenthood, and hence facilitating educational groups using different types of experiential methods should be recommended.^44^ Women in our study were also very reliant on the written information provided to them by their healthcare providers. Hence, health services should produce up‐to‐date user‐friendly online and paper‐based resources or materials for women and encourage engagement with such materials from the healthcare professionals' perspective to support discussion with patients. However, given the diversity in which women prefer to receive information, it is important to tailor and utilize diverse types of intervention approaches to maximize engagement and effectiveness of antenatal interventions.

Healthcare professionals should engage in active discussions with women, regarding risk factors for stillbirth. Antenatal education standards and healthcare professionals' training programmes should guide to support healthcare professionals in promoting health with their patients, and also be able to discuss risk factors for stillbirth or other potential adverse outcomes. The National Women and Infant Health Programme developed the National Standards for Antenatal Education in Ireland in 2020.^44^ Although this document highlights the importance of including an element of better health and well‐being (Theme 4) in the optimal standards of antenatal education, this theme only refers to supporting healthy habits and addressing mental health concerns generally, without specifying the need to address issues such as the risk factors for stillbirth with women.^44^

Previous research has also demonstrated that stillbirth is a taboo topic in society.^29^, ^45^, ^46^ However, as seen from our results, most women would welcome information about stillbirth and risk factors as a tool to improve their health and prevent it from happening to them, or even as emotional preparation after experiencing a stillbirth. However, a high number of women also acknowledged that receiving this information would have the potential to increase their levels of anxiety and concerns, which is an issue that should be taken into consideration. Literature in areas such as sexual health or weight management has shown that healthcare professionals are reluctant to discuss topics perceived as taboo or difficult with their patients,^47^ and stillbirth is presumably a similarly unmentionable topic.^45^ We believe that it is important that official bodies regulating antenatal education standards in different high‐income countries include specific guidance regarding advising women about health habits, risk factors and potential adverse outcomes such as stillbirth to support healthcare professionals in providing antenatal education with confidence. Furthermore, as mentioned by multiple women, receiving this information has the potential to increase their anxiety levels or their concerns, and hence, healthcare professionals are prepared to address women's concerns and resolve their doubts, trying to alleviate their worries by providing accurate and sensitive information. The different types of pregnancy loss have different implications regarding care, and hence, breaking the silence around stillbirth is essential to ensure that women who experience stillbirth received the appropriate support.^29^

Our study has some strengths and limitations. The study's first limitation is related to the sample of interviewed women. The study participants were predominantly White, educated, heterosexual women from developed countries, and, likely, their experiences with behaviour change during pregnancy and challenges identified are not generalizable to people from different sociodemographic backgrounds. Additionally, the rates of smoking and alcohol consumption were also lower in our sample than in the general pregnant population, thus findings related to this modifiable risk factor for stillbirth are likely influenced by this. Further, another limitation that we encountered when conducting the interviews is that in some instances women struggled to recall some parts of their experiences, given the timeframe in which they were interviewed. This might also be explained by the changes in memory that occur during pregnancy and have been described in the literature before, where women reported memory deficits categorized as a general sense of ‘fogginess’.^30^ The number of women approached to consent to this study was high for a qualitative design. This decision was made to ensure that we were able to obtain an appropriate amount of rich data and anticipate that most women might decline to participate a few months after leaving the hospital. Of the 44 women recruited, only 18 participated in the interviews, indicating that possibly our timeframes did not suit the majority of women and highlighting the challenges of recruiting women during the busy postnatal period. Furthermore, given that only the clinical team has access to the patient's medical record, we are unaware of how many women were approached by the clinical recruiting team and declined to participate as this number was not recorded. The researchers only obtained the contact information of the women who consented to participate in the study. This hinders the assessment of the efficacy of our recruitment method, and it also limits our knowledge about potential differences between women who agreed to participate and women who declined. It is possible that women who chose to take the time to participate in this study were adapting better to the postpartum period than those who could not participate. This might be related to their support networks, their baby's sleep patterns or temperament, their baby's health needs or their own health needs. Hence, it is important to acknowledge that women participating in this study might have been having a more positive experience regarding their pregnancy, birth and the postpartum period, and so these factors also need to be considered when trying to generalize these findings.

Despite these limitations, our study also has several strengths. Our inclusion and exclusion criteria were designed to recruit women with uncomplicated pregnancies and births, enabling the exploration of experiences of women receiving the most ‘generalised’ type of care in our maternity services; this increases the generalizability and transferability of our findings to other low risk/uncomplicated pregnancies in similar maternity systems. Furthermore, the use of online interviews was praised by some of the women as a very comfortable way to engage in research, without causing much interference in their normal lives. To date, there is only one other study exploring women's perception of public health messages to reduce stillbirth, in this case, exploring the views of migrant women in the United Kingdom.^43^ The findings of this study are similar to our findings in that they highlight the complexities of discussing stillbirth with women during their pregnancies and the importance of developing culturally appropriate resources to secure efficient communication.

## 5 CONCLUSION

This study aimed to explore women's experiences of modifiable risk factors during pregnancy, awareness of stillbirth and its risk factors, as well as their experience with information provision during their antenatal care. The findings of this study have shown that women with uncomplicated pregnancies receive very poor information about health behaviours, behavioural risk factors or stillbirth during pregnancy. Women have high levels of understanding of how to have a healthy pregnancy, but the link between behavioural risk factors and potential outcomes such as stillbirth is not considered. Information provision during antenatal care was not sufficient, and women had to rely upon their information‐seeking behaviour. Most women perceived receiving information about stillbirth during antenatal care to be useful to help preventive efforts, although others acknowledge the potential for this information to raise some concerns highlighting the importance of using sensitive nonjudgemental language.

Information provision alone is not sufficient to support behaviour change, however, it might act as a first step to engage in discussions and facilitate women seeking adequate care for their specific needs. Healthcare professionals should break the silence around stillbirth and incorporate risk factors, health habits and stillbirth in their routine discussions with women, especially in terms of outcomes for their babies, to motivate women to engage in behaviour change. Tackling the modifiable maternal risk factors for stillbirth by providing information and supporting women with behaviour change during pregnancy might contribute to reducing the stillbirth rates in Ireland.

## CONFLICT OF INTEREST

The authors declare no conflict of interest.

## Supporting information

Supporting information.

Supporting information.

## Data Availability

The data that support the findings of this study are available on request from the corresponding author. The data are not publicly available due to privacy or ethical restrictions.
