# Supplementary material for: Exploring first‐time mothers' experiences and knowledge about behavioural risk factors for stillbirth
Source: Health Expect. 2022 Nov 23;26(1):329–42. doi: 10.1111/hex.13662 (PMC9854314; doi:10.1111/hex.13662)
Supplement: Supplementary file 1 — Supporting information. [file HEX-26--s002.docx]

| Topic | Item | Page |
| --- | --- | --- |
| Title | | |
| S1 Title | Concise description of the nature and topic of the study Identifying | Page 1.  Study title “Exploring first time mothers’ experiences and knowledge about behavioural risk factors for stillbirth” |
| S2 Abstract | Summary of key elements of the study using the abstract format of the intended publication; typically includes background, purpose, methods, results, and conclusions | Page 1. |
| Introduction | | |
| S3 Problem formulation | Description and significance of the problem/phenomenon studied; review of relevant theory and empirical work; problem statement | Introduction Paragraph 1: Description of problem/phenomenon.  Introduction Paragraph 2 and 3: Review of previous literature.  Introduction Paragraph 4: problem statement and study aims. |
| S4 Purpose or research question | Purpose of the study and specific objectives or questions | Introduction Paragraph 4: study aim.  *The main objective of this study was to explore women’s experiences of modifiable factors during pregnancy and knowledge and beliefs regarding behavioural risk factors related to stillbirth. Additionally, we aimed to examine women’s experiences, if any, of being informed about such risk factors during their antenatal care.* |
| Methods | | |
| S5 Qualitative approach and research paradigm | Qualitative approach (e.g., ethnography, grounded theory, case study, phenomenology, narrative research) and guiding theory if appropriate; identifying the research paradigm (e.g., postpositivist, onstructivist/interpretivist) is also recommended; rationale | Methods section, 2.1 Design. |
| S6 Researcher characteristics and reflexivity | Researchers’ characteristics that may influence the research, including personal attributes, qualifications/experience, relationship with participants, assumptions, and/or presuppositions; potential or actual  interaction between researchers’ characteristics and the research  questions, approach, methods, results, and/or transferability | Methods section,2.7 Reflexivity statement. |
| S7 Context | Setting/site and salient contextual factors; rationale | Methods section. 2.3 Setting and Sample. |
| S8 Sampling strategy | How and why research participants, documents, or events were selected; criteria for deciding when no further sampling was necessary (e.g., sampling saturation); rationale | Methods section. 2.2 Recruitment and 2.4 Data collection. |
| S9 Ethical issues pertaining to human subjects | Documentation of approval by an appropriate ethics review board and participant consent, or explanation for lack thereof; other  confidentiality and data security issues | Methods, section 2.6. Ethical considerations |
| S10 Data collection methods | Types of data collected; details of data collection procedures including  (as appropriate) start and stop dates of data collection and analysis,  iterative process, triangulation of sources/methods, and modification  of procedures in response to evolving study findings; rationale | Methods. Section 2.4 Data collection. |
| S11 Data collection instruments and technologies | Description of instruments (e.g., interview guides, questionnaires) and devices (e.g., audio recorders) used for data collection; if/how the  instrument(s) changed over the course of the study | Methods. Section 2.4 Data collection and supplementary file 2. |
| S12 Units of study | Number and relevant characteristics of participants, documents, or  events included in the study; level of participation (could be reported  in results) | Results. Section 3.1 Sample Characteristics and Table 1. |
| S14 Data analysis | Process by which inferences, themes, etc., were identified and developed, including the researchers involved in data analysis; usually  references a specific paradigm or approach; rational | Methods. Section 2.5 Data analysis |
| S15 Techniques to enhance trustworthiness | Techniques to enhance trustworthiness and credibility of data analysis (e.g., member checking, audit trail, triangulation); rationale | Methods. Section 2.5 Data analysis.  “One researcher (X) read and re-read all of the interview transcripts. Deductive open coding was then initiated which facilitated the identification of units of meanings that related to the research aims. Subsequently, those codes were categorised and grouped into themes, and re-labelled where appropriate. A record of the evolvement of the themes and the category names was always kept. Further analysis allowed the researchers (TES, KMS) to group the different categories into themes, by refining its meaning to portray the story the data tells. A second author (KMS) reviewed and followed the coding process at all stages, and discussions were held as necessary.” |
| Results/findings | | |
| S16 Synthesis and interpretation | Main findings (e.g., interpretations, inferences, and themes); might  include development of a theory or model, or integration with prior  research or theory | Results. 3.2 Findings section. |
| S17 Links to empirical data | Evidence (e.g., quotes, field notes, text excerpts, photographs) to  substantiate analytic findings | Results. 3.2 Findings section (quotes)). |
| Discussion | | |
| S18 Integration with prior work, implications,  transferability, and contribution(s) to the field | Short summary of main findings; explanation of how findings and conclusions connect to, support, elaborate on, or challenge conclusions of earlier scholarship; discussion of scope of application/generalizability; identification of unique contribution(s) to scholarship  in a discipline or field | Discussion.  Paragraph one includes a short summary of findings.  The rest of the conclusion addresses all the rest of items. |
| S19 Limitations | Trustworthiness and limitations of findings | Discussion. Paragraph 8. |
| Others | | |
| S20 Conflicts of interest | Potential sources of influence or perceived influence on study conduct  and conclusions; how these were managed | This information can be found at the bottom of the paper. |
| S21 Funding | Sources of funding and other support; role of funders in data  collection, interpretation, and reporting | This information can be found at the bottom of the paper. |
