# Supplementary material for: Exploring first‐time mothers' experiences and knowledge about behavioural risk factors for stillbirth
Source: Health Expect. 2022 Nov 23;26(1):329–42. doi: 10.1111/hex.13662 (PMC9854314; doi:10.1111/hex.13662)
Supplement: Supplementary file 2 — Supporting information. [file HEX-26--s001.docx]

| Introduction |  |
| --- | --- |
| History and health habits | Did you do anything to prepare to get pregnant?  What were your main worries related to health? Why?  What habits did you have to change when you became pregnant?  How difficult/easy did you find..?  Why did you make those decisions?  If a friend were to get pregnant, what would you recommend so she can make sure she has a healthy pregnancy? |
| Risk factors for stillbirth | Are you aware of any maternal behaviours that might be bad for the baby?  And do you know any pregnancy issues that might be associated with those behaviours?  What do you know about smoking/alcohol/drugs during pregnancy?  What do you know about diet or physical activity during pregnancy?  Did you learn how to manage your weight gain safely during pregnancy?  What do you know about sleep position?  Where did you get the information?  Who approached you with this information?  Have you heard of stillbirth? Can you explain it to me? When do you think it can happen?  Have you heard of any behaviours that might increase the risk of stillbirth?  Did you discuss any particular behaviours with in your antenatal care? (smoking/alcohol/drugs/attendance at antenatal care/sleep position/weight management, diet exercise) |
| Feelings and opinion | Were you approached by your doctor/midwife to talk about stillbirth? or risk factors for stillbirth?  How did you feel (how would have made you feel) when your doctor/midwife talked to you about stillbirth?  Do you think it would have been useful for your doctor/midwife to talk to you about stillbirth? or the risk factors for it?  What benefits could pregnant women (did you) get from discussing these risks with their healthcare providers?  How or when would you have liked the doctor/nurse to approach the subject?  (prompts: What gestational age? What opportunity? e.g: routine clinic, in Emergency Department for a problem, etc.?)  Do you think it is important to speak about the risk of stillbirth to a healthy low risk pregnant woman? |
| Information sources | Among all of the sources available, which one do you trust the most?  How did you use that source of information?  What did you like about that source of information?  Did you receive or find any conflicting information? (prompts: internet, GP, midwife, obs., family, fiends) About what? |
| Intervention | Have you found anything that helped you promote or support your health habits while you were pregnant? For example, text messages with health related information; educational groups; one on one sessions with healthcare professionals, etc.?  Can you describe it to me? What elements of it did you find most helpful?  Do you think you had anyone in your social environment that influenced your health during your pregnancy? How?  In your opinion, what kind of support or information service would help pregnant women to improve their health habits?  What are the best ways to provide this information or who should be providing it?  (Prompts: information sessions, leaflets, websites, one to one sessions, suggestions?)  When is the best time to deliver this intervention to women? When would you have liked to get the information? |
